# Hybrid neoplasm of the lacrimal gland, adenocarcinoma NOS with primary squamous cell carcinoma; A rare case report

**DOI:** 10.1016/j.jobcr.2025.07.025

**Published:** 2025-08-07

**Authors:** Gopikrishnan Vijayakumar, Anjali Narwal, Mala Kamboj, Garima Rawat

**Affiliations:** a Department of Oral Maxillofacial Pathology and Microbiology, Post Graduate Institute of Dental Sciences (PGIDS), Rohtak, Haryana, 124001, India; b Department of Pathology, Dharamshila Narayana Super Speciality Hospital, New Delhi, India

**Keywords:** Adenocarcinoma NOS, Hybrid neoplasm, Primary ductal adenocarcinoma, Squamous cell carcinoma

## Abstract

A hybrid neoplasm is the coexistence of two histologically distinct neoplasms which arise in the same topographical area resulting in a single common mass. A seventy-one-year-old female presented with a complaint of loss of vision and pain in her right eye for a period of five months. On clinical-radiological investigations, a single ovoid,non-fluctuant, firm, tender swelling was present on the right supraorbital region with mild proptosis and redness of the right eye. Magnetic resonance imaging showed a well-defined, lobulated heterogeneously enhancing lesion on the superolateral part of the right orbit suggestive of lacrimal gland neoplasm. The specimen was submitted after wide excision with orbital exenteration and ipsilateral radical neck dissection. Histopathological evaluation revealed two different morphologic patterns of infiltrating islands of epithelial cells suggestive of an adenocarcinoma and squamous carcinoma. Histopathology with additional immunohistochemical workup concluded the lesion to be a Hybrid neoplasm of the Lacrimal gland; Adenocarcinoma NOS with Primary squamous cell carcinoma. Hybrid neoplasm of the head and neck region is found to be common in salivary glands while its occurrence in the lacrimal gland is very rare in literature.

## 1 Introduction

Lacrimal gland neoplasms comprise up to 18 % of all orbital neoplasms, of which 70–75 % are epithelial in origin. More than 70 % of lacrimal gland epithelial neoplasms are pleomorphic adenomas, while 25 % are malignant tumours.^1^ Due to the histological and immunological similarities, these tumours are similar to those occurring in the salivary glands.^1^^,^^2^ Majority of the malignant lesions in lacrimal gland are represented by adenoid cystic carcinomas (ACC)(13.4 %) and carcinomas-ex-pleomorphic adenomas (CaXPA) and few cases of basal cell adenocarcinoma (BAC), primary ductal adenocarcinoma (PDA), acinic cell carcinoma, mucoepidermoid carcinoma (MEC)(3.6 %),^3^ oncocytic carcinoma, polymorphous adenocarcinoma (PAC) and myoepithelial carcinoma.^1^, ^2^, ^3^, ^4^

Hybrid tumour or hybrid carcinoma is a very rare neoplasm first reported in English literature by Ballestin et al. (1996) in salivary glands.^5^ These neoplasms are composed of two separately different histopathological entities, each one of which conforms to an exactly defined tumour category, arising within the same topographical area.^5^^,^^6^ The incidence of hybrid tumours in the head and neck is reported to be much reported with salivary glands, but only two cases were documented in the lacrimal gland. The first case was squamous cell carcinoma with ductal adenocarcinoma, and the second case of squamous cell carcinoma with epithelial myoepithelial carcinoma.^7^ In the present case, we report a hybrid neoplasm of adenocarcinoma NOS with squamous cell carcinoma in the lacrimal gland.

## 2 Case presentation

A seventy-one-year-old female having no comorbidities presented with a complaint of loss of vision and pain in her right eye for five months. On examination, the patient was moderately built with Eastern Cooperative Oncology Group (ECOG) performance status-2 with no associated history of fever, fatigue, diabetes, hypertension, weight loss or other similar swellings elsewhere. There was no personal or family history related to the present condition. The routine laboratory findings were within normal limits for the age. Medical history of irritation, continuous radiating pain, excessive lacrimation and gradual reddening of the right eye with no other systemic diseases.

Clinical examination revealed a single ovoid non-fluctuant firm tender swelling of size 4 × 3 cm on the right supraorbital region with mild proptosis and redness of the right eye. Magnetic resonance imaging revealed a well-defined, lobulated heterogeneously enhancing lesion on the superolateral part of the right orbit involving the right superior, lateral recti abutting the superior oblique muscle, causing mild proptosis and scalloping on the superolateral wall of the orbit, clinically suggestive of lacrimal gland neoplasm. Specimen after wide excision with orbital exenteration and ipsilateral radical neck dissection was submitted for histopathological evaluation. The gross specimen measured approximately 6.5 × 6x5 cm with a lesion measuring 3.4 × 3.2 × 3.1 cms with excised posterior and superolateral bony orbital margins was received and processed.

**Fig. 1:**
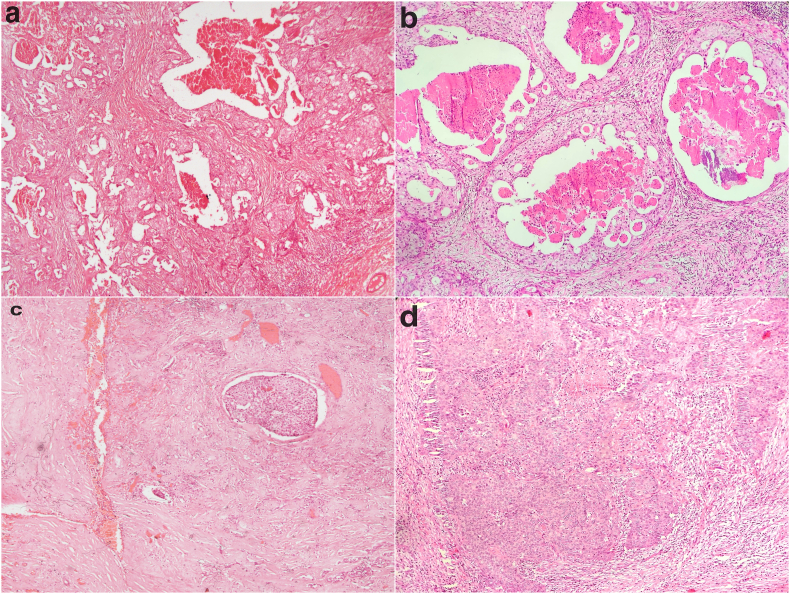
Photomicrograph **1a:** Photomicrograph showing infiltrating tumour islands with ductal architecture. Tumour epithelial cells with hyperchromatic vesicular nuclei and eosinophilic cytoplasm arranged in a ductal pattern (H&E ×40). **1b:** Tumour islands showing areas with Roman bridge pattern and central comedo necrosis (H&E ×200). **1c:** Tumour cells with a scirrhous pattern of infiltrating tumour cells with abundant hyalinised stroma (H&E ×40). **1d:** Photomicrograph showing invading sheets of pleomorphic epithelioid cells without any ductal/lobular architecture. Tumour cells are infiltrating polygonal cells with moderate pleomorphic vesicular nuclei and abundant eosinophilic cytoplasm (H&E ×100).

Multiple sections sampled from the primary tumour mass on histopathological examination revealed infiltrating islands of epithelial cells with hyperchromatic vesicular nuclei and eosinophilic cytoplasm arranged in ductal architecture (Fig. 1a). Tumour islands showed areas with a Roman bridge pattern and central comedo necrosis suggestive of an adenocarcinoma (Fig. 1b). Few areas had scirrhous patterns of infiltrating tumour cells with abundant hyalinised stroma (Fig. 1c). Brisk mitosis (>12/10 high power field) and perineural invasion in large nerves were also evident. Towards another focus within the primary tumour, separate from the adenocarcinoma region by collagenous stroma, invading sheets of pleomorphic epithelioid cells without any ductal/lobular architecture were found which morphologically varied from the adenocarcinoma pattern. These cells were of polygonal epithelioid morphology with pleomorphic vesicular nuclei and abundant eosinophilic cytoplasm (Fig. 1d). Multiple cervical lymph nodes at right levels II, III and V showed metastatic deposits of adenocarcinoma. The marginal bone resected from the superolateral orbital wall showed infiltration by adenocarcinoma but with safe bone margins. Sections from the eyeball, optic nerve cut end, choroid, and anterior and posterior chambers were free of tumour. The resected skin and soft tissue margins, extraocular muscles and posterior bony orbital wall were free of any tumour infiltration.

**Fig. 2:**
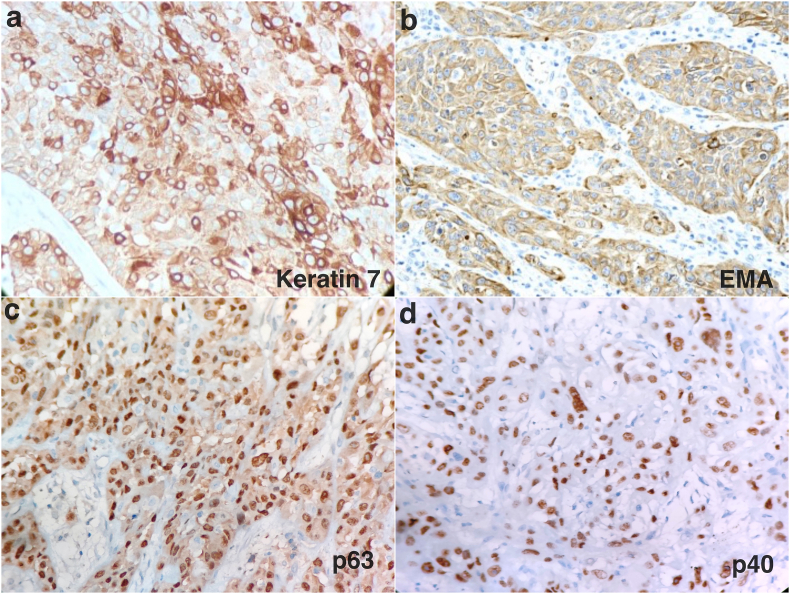
Photomicrograph showing immunohistochemical markers (a) diffuse strong positive Keratin 7 in tumour cells in adenocarcinoma morphology, (b) diffuse strong positive EMA in epithelioid tumour cells without ductal morphology (c) diffuse strong positive p63 in epithelioid tumour cells without ductal morphology, (d) diffuse positive p40 in epithelioid tumour cells without ductal morphology.

On immunohistochemical analysis, the adenocarcinoma cells showed diffuse strong immunopositivity for Keratin 7 while immunonegativity for EMA, Keratin 5/6, p63, p40, Androgen receptor (AR), gross cystic disease fluid protein 15 (GCDFP-15), Her2Neu, BerEp4, S100, SMA and calponin. The tumour cells in areas with pleomorphic polygonal epithelioid type cells spread in sheets without ductal morphology showed diffuse strong immunopositivity for EMA,p63 and p40 but immunonegative for Keratin 7, AR, GCDFP-15, Her2Neu, BerEp4, S100, SMA and calponin (Fig. 2a–d).

## 3 Discussion

Lacrimal gland tumours are described as analogous to the salivary gland counterparts namely ductal adenocarcinomas, adenoid cystic carcinomas, pleomorphic adenoma, and carcinoma ex pleomorphic adenoma.^1^, ^2^, ^3^ Primary ductal adenocarcinoma (PDA)/Adenocarcinoma not otherwise specified (NOS)are currently classified under adenocarcinoma of the lacrimal gland which histologically and immunologically resemble the salivary duct adenocarcinoma (SDC)and invasive breast cancer.^3^^,^^7^^,^^8^ PDA constitutes 2 % of all epithelial lacrimal gland tumours while adenocarcinoma NOS incidence is up to 8.4 % but with similar clinical presentations.^3^ Histologically, both PDA and adenocarcinoma NOS are high-grade, aggressive malignant epithelial tumours with glandular formation in duct-like configuration and extensive comedo necrosis. The tumour cells have eosinophilic to granular cytoplasm and large vesicular nuclei, abundant mitosis.^3^^,^^7^, ^8^, ^9^ Areas with abundant hyalinization and infiltrating tumour islands resembling a scirrhous pattern are also described in the literature.^7^^,^^10^ The adenocarcinoma NOS is a diagnosis of exclusion and is differentiated from PDA by the positive immunohistochemical expression of the latter by androgen receptor (AR) and gross cystic disease fluid protein 15 (GCDFP-15).^3^

In the present case, adenocarcinoma cells were immunoreactive only for low molecular weight cytokeratin- Keratin 7 while immunonegative for all other epithelial markers like EMA,p63 and high molecular weight cytokeratin-Keratin5/6. The possibility of a metastatic breast adenocarcinoma was ruled out with an immunonegative BerEp4 and Her2neu expression. Primary ductal adenocarcinoma (PDA) was ruled out as Androgen receptor (AR) and GCDFP-15 were negative in the present tumour cells favouring a diagnosis of adenocarcinoma NOS than PDA in which 90–95 % cases are reported to be positive for these markers.^3^

Few areas exhibiting invading strands of pleomorphic tumour cells with abundant hyalinization mimicked a focus of carcinoma ex pleomorphic adenoma. But the absence of any histologic focus of pre-existing pleomorphic adenoma or history of any pre-existing tumour ruled out the possibility of an adenocarcinoma ex pleomorphic adenoma.^7^^,^^10^ The invading sheets of pleomorphic polygonal tumour cells without ductal morphology showed diffuse strong immunoreactivity for p40 and p63 and immunonegativity for Keratin 7. This histomorphologic and immunohistochemical presentation was a diagnostic dilemma between myoepithelial carcinoma and squamous cell carcinoma. These tumour cells showed diffuse immunoreactivity to EMA and immunonegativity to myoepithelial markers like S100, SMA and calponin. A diffuse strong positivity for EMA, p63 and p40 with immunoreactivity for other myoepithelial markers favoured a primary squamous cell carcinoma over myoepithelial carcinoma. These squamous cell carcinoma components were present as a histologically separate entity from the adenocarcinoma areas and not intermixed, ruling out the possibility of adenosquamous carcinoma which is a variant of squamous cell carcinoma. Based on the histopathological and immunohistochemical analysis, the present case was reported as a primary tumour of the lacrimal gland with a hybrid presentation of coexisting adenocarcinoma NOS and squamous cell carcinoma (Table 1).

**Table 1:** Table of differential diagnosis and immunohistochemistry for diagnosis. IHC panel used.

Marker	Expression in areas with ductal differentiation/Adenocarcinoma areas	Expression in areas without ductal differentiation
Keratin 5/6	Negative	Negative
Keratin 7	Positive	Negative
EMA	Negative	Positive
P63	Negative	Positive
P40	Negative	Positive
AR	Negative	Negative
GCDFP-15	Negative	Negative
BerEp4	Negative	Negative
Her2Neu	Negative	Negative
S100	Negative	Negative

Differential diagnosis of in areas with ductal differentiation/Adenocarcinoma areas	Primary ducal adenocarcinoma PDA	Adenocarcinoma NOS
IHC for diagnosis	90 % cases are positive for AR, GCDFP-15, Her2neu	Diagnosis- By exclusion Mostly negative for AR GDFP-15, Her2neu (EXCLUDE-PDA, Ca ex PA, MEC, PAC)
**Inference**	The IHC panel favours diagnosis of **Adenocarcinoma NOS**	

Differential diagnosis of in areas without ductal differentiation	Myoepithelial carcinoma	SQUAMOUS CELL CARCINOMA
IHC for diagnosis	Positive –p40,p63 Negative AR,GCDFP15,Her2 Neu, BerEp4,	Positive – EMA,p40,p63
		Negative –S100, SMA, Calponin
Inference	The IHC panel favours diagnosis of **Squamous cell carcinoma**	

The tumour staging was pT2cN1M0 as per the 8th ed American Joint Cancer Committee (AJCC) staging of lacrimal gland carcinomas based on the most aggressive adenocarcinoma component. The nodal metastatic deposits were found to be only adenocarcinoma. The patient is kept on regular follow-up after prosthetic rehabilitation of the right eye and is disease free for the past one year.

## 4 Conclusion

The pathophysiology for hybrid presentation is still debated, as it can either occur denovo or by a high-grade transformation in the pre-existing low-grade tumour. The cervical node metastases of hybrid lesions may consist wholly or predominantly of one subtype the prognosis is dependent on the higher-grade subtype.

## Author contributions

All authors were equally involved in diagnosis of case, case study design, investigation of literature, and writing the paper.

## Ethical approval

No patient identifiers or details are mentioned in the manuscript. This article does not contain any studies with animals performed by any of the authors. All procedures performed in this study involving human participants were in accordance with the ethical standards of the institutional and/or national research committee and with the 1964 Helsinki declaration and its later amendments or comparable ethical standards.

## Patient and ethical approval

This article does not contain any studies with animals performed by any of the authors. All procedures performed in this study involving human participants were in accordance with the ethical standards of the institutional and/or national research committee and with the 1964 Helsinki declaration and its later amendments or comparable ethical standards.

## Patient and ethical approval

No patient identifiers or details are mentioned in the manuscript. Informed signed consent obtained before all treatment procedures.

## Source of funding

None.

## Declaration of competing interest

The authors declare that they have no known competing financial interests or personal relationships that could have appeared to influence the work reported in this paper.
